# Depression and diagnosis of neurocognitive impairment in HIV-positive patients

**DOI:** 10.1590/1414-431X20165344

**Published:** 2016-09-12

**Authors:** C.A.T. Pinheiro, L.D.M. Souza, J.V.S. Motta, E.F. Kelbert, M.S. Souza, C.S.R. Martins, F.M.C. Coelho, K.A.T. Pinheiro, R.T. Pinheiro

**Affiliations:** 1 Serviço de Assistência Especializada, Faculdade de Medicina, Universidade Federal de Pelotas, Pelotas, RS, Brasil; 2 Programa de Graduação em Saúde e Comportamento, Universidade Católica de Pelotas, Pelotas, RS, Brasil

**Keywords:** AIDS, Depression, Neurocognitive, HIV, HAND, CD4

## Abstract

Neurocognitive impairment (NCI) is frequently observed in patients infected with human immunodeficiency virus (HIV) and results from the compromise of subcortical brain structures by the virus. The manifestations of NCI range from asymptomatic impairment to dementia. In addition to cognitive impairment resulting from HIV infection, other factors such as depression are associated with the loss of cognitive functions. The aim of this study was to estimate the prevalence of NCI in HIV-positive patients in a city in southern Brazil and to establish possible associations for the prevalence of NCI with HIV-related and other risk factors. This cross-sectional study of HIV-positive outpatients was conducted in a specialized care service in the city of Pelotas in Southern Brazil. Sociodemographic data and HIV-related information were collected, and all patients underwent psychiatric and neurocognitive evaluations. The prevalence of NCI among the 392 patients was 54.1% when tracked using the IHDS (International HIV Dementia Scale) and 36.2% when the IHDS was associated with a battery of complementary tests. A bivariate analysis suggested an association of NCI with gender, age, educational level, depression, current CD4 count and lowest CD4 count. The association of NCI with depression remained in the Poisson regression (PR=1.96, 95%CI=1.12-3.42). The prevalence of cognitive impairment in HIV-positive patients estimated in this study is in accordance with international and Brazilian data. Of the factors analyzed, depression showed the greatest evidence of association with neurocognitive loss. Based on our findings, the inclusion of instruments to evaluate depression in our services for patients with HIV and acquired immunodeficiency syndrome (AIDS) is recommended.

## Introduction

The human immunodeficiency virus (HIV) is neurovirulent (1,2) and frequently causes brain impairment. Subcortical brain structures are the regions most often affected by HIV, and the resulting changes to these structures cause deficits in attention, learning, memory, information processing speed and problem-solving ability (2). According to norms established by the HIV Neurobehavioral Research Center, these HIV-associated neurocognitive disorders (HAND) are classified into the following three conditions: asymptomatic neurocognitive impairment, mild neurocognitive disorder, and HIV-associated dementia (3).

Data on the prevalence of HAND vary greatly. Following the American Association of Neurology's establishment of HIV-related cognitive impairment diagnostic criteria in 2007, studies have reported a prevalence of 30–60% (4–6). There are few data on the prevalence of these disorders in Brazil (7).

One of the difficulties associated with establishing the true prevalence of HAND is the lack of user-friendly diagnostic tools for use in clinical practice (6,8). In an attempt to solve this problem, a screening instrument known as the International HIV Dementia Scale (IHDS) (9) was created to identify neurocognitive impairment (NCI) in HIV-positive patients. The IHDS is a rapid screening test that has been used in populations in the United States and Uganda and shows high sensitivity (80% for both populations) and specificities of 57 and 55%, respectively, for a cut-off point of ≤10 on a scale ranging from 0 to 12 points. This scale was recently validated in Brazil by Rodrigues et al. (10), who found a sensitivity of 78.5% and a specificity of 80.8% in the identification of HIV-related dementia. This validation study revealed a prevalence of HAND of 52.4%. In a study by Troncoso et al. (11) conducted in Marília, SP, Brazil, using the IHDS, the prevalence of HAND was 53.2%. In addition, in the city of Recife, PE, Brazil, Arraes (12) diagnosed 67.3 and 33.7% of individuals with HAND using the IHDS with cut-offs of ≤11 and ≤10, respectively.

The combination of multiple simple instruments for the evaluation of cognitive impairment has been proposed to increase the sensitivity and specificity of HAND diagnosis. Skinner et al. (8) compared the performances of various neuropsychological tests, including the Color Trails and Grooved Pegboard tests. In the multicenter study by Wright et al. (13), which included patients from Brazil, Australia, North America and Thailand, a battery of five tests, including the Grooved Pegboard, Finger Tapping, Color Trails 1 and 2 and Timed Gait tests, was used for HAND diagnosis. These tests are easy to perform and do not present any language or cultural limitations.

Many factors, including the duration of HIV infection, the lowest CD4 count and psychiatric disorders, have been associated with HAND (14). Among the associated psychiatric disorders, depression is often diagnosed in patients with HIV or acquired immunodeficiency syndrome (AIDS) (15) at a prevalence of 12–66% (14–17). Studies conducted in Brazil have estimated a prevalence of 32–34% (16,18). The study by Passo et al. (18), which was conducted in Pelotas, RS, Brazil, showed a high risk of suicide (34.1%).

The main objective of our study was to estimate the prevalence of cognitive impairment and associated factors in a city in Southern Brazil using the IHDS, Grooved Pegboard Test, Color Trails Tests 1 and 2, Finger Tapping Test, and the Montreal Cognitive Assessment (MoCA) test.

## Material and Methods

This cross-sectional study collected data from HIV-positive patients aged at least 18 years who were diagnosed according to the Brazilian Ministry of Health protocols (19) and attended consultations in the Serviço de Assistência Especializada (SAE), in the city of Pelotas in Southern Brazil in 2015. Patients with prior neurological illness and/or psychotic psychiatric disorders, with current or previous opportunistic infections of the central nervous system, those with a history of chronic neurological disorder not related to HIV, those with active psychiatric disorder, those who suffer from alcoholism, and those with physical deficiencies that could interfere with the tests, such as blindness, significant hearing loss, and amputations, were excluded.

All patients attending the service were invited to participate in the study. Those who agreed to participate were asked to sign an informed consent form. The protocol was approved by the Universidade Católica de Pelotas Ethics Committee. The participants answered a sociodemographic questionnaire and underwent psychiatric and neurocognitive evaluations. The MINI-International Neuropsychiatric Interview (MINI-Plus) was used for the psychiatric evaluation. The Brazilian Portuguese version of this instrument has been found to be useful for the diagnosis of psychiatric disorders in research and clinical practice (20). The neurocognitive evaluation was carried out with the following tools: Grooved Pegboard Test, Color Trails Tests 1 and 2, Finger Tapping Test, MoCA, and the IHDS. A cut-off score of 10 or less was used for the latter. All evaluations were performed at the SAE.

Various clinical aspects of the patients were determined through the analysis of laboratory tests and the patient's medical record data. Information on the stage of HIV infection, use of antiretroviral therapy (ART), treatment time, diagnosis time, CD4 count, viral load and comorbidities was collected.

In Brazil, the only evaluation tool with a standardized measure for defining a cut-off is the IHDS. Therefore, the scores of the other tests (MoCA, Color Trails Test 1 and 2, Finger Tapping Test, Grooved Pegboard Test) were divided into quartiles, and in an attempt to obtain a greater specificity, individuals with scores in the upper quartile for at least three of the five measures and who reached the IHDS cut-off value were defined as being positive for NCI.

A descriptive analysis of sociodemographic and clinical data was conducted, and the frequency of each categorical variable and the mean and standard deviation of each continuous variable were calculated. A bivariate analysis was performed considering the effect measures, and associations were assessed. The *chi*-square test for proportions and the independent *t*-test with the Levene correction for continuous variables were used. Hierarchical Poisson regression models were constructed using Stata 12.0 software (StataCorp LP, USA).

For Poisson regression, the theoretical model assumes a hierarchy of levels in relation to the outcome, i.e., more distal variables determine NCI. In this study, the model was divided into two levels. The first level included gender, age, educational level and skin color, and the second level comprised alcohol dependence, depression, social phobia, manic episodes, suicide risk, obsessive compulsive impairment, abuse of illicit drugs, years since diagnosis, administration of the first ART regimen, withdrawal from ART in the last 3 months, initial viral load, current viral load, baseline CD4 count, recent CD4 count, and lowest CD4 count. The variables included in the model had a P value of <0.20 in the crude analysis, and variables with P values less than 0.05 after adjustment were retained in the analysis.

## Results

A total of 434 patients were evaluated, and 392 of these patients were selected for analysis. Of the selected patients, 55.4% were female, and their mean age was 42±11.58 years (range 18–82 years). Seventy-six percent of the patients had a mean educational level of less than 8 years (Table 1). Regarding disease staging, 34.3% met the criteria for AIDS diagnosis, and 58.5% had asymptomatic infection. Of the included patients, 89.3% were using ART. In addition, 74% of the patients using ART had a viral load of less than 50 copies, and 84.1% had a CD4 count greater than 200 cells/mm^3^. Forty-two patients were excluded due to lack of data in their medical records, not having completed the battery of tests, or refusal to participate. The characteristics of these patients were similar to those included in the analysis.

**Table:**
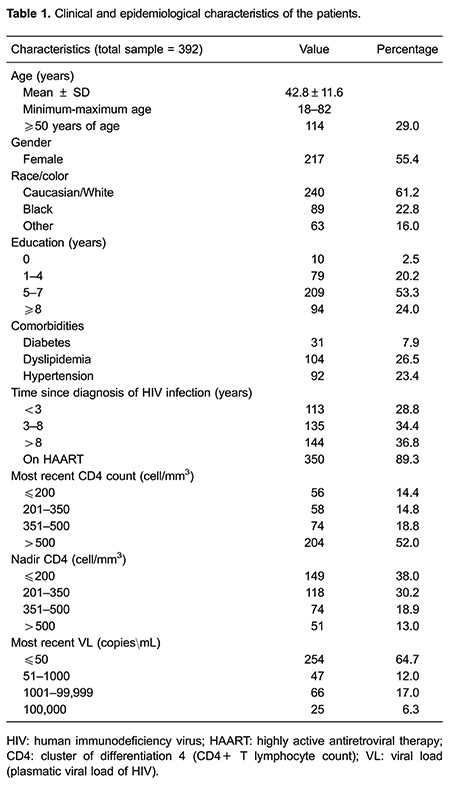

The prevalence of NCI among the 392 patients was 54.1% when assessed using the IHDS and 36.2% when the IHDS was associated with the complementary neurological evaluation tests.

Taking into account the patients who screened positive on the IHDS and with scores in the upper quartile for at least three tools in the battery of neurocognitive tests, the bivariate analysis showed an association with the following variables: gender, age, race, educational level, depressive episode, use of ART, last CD4 count and nadir CD4. Results are presented in Table 2.

**Table:**
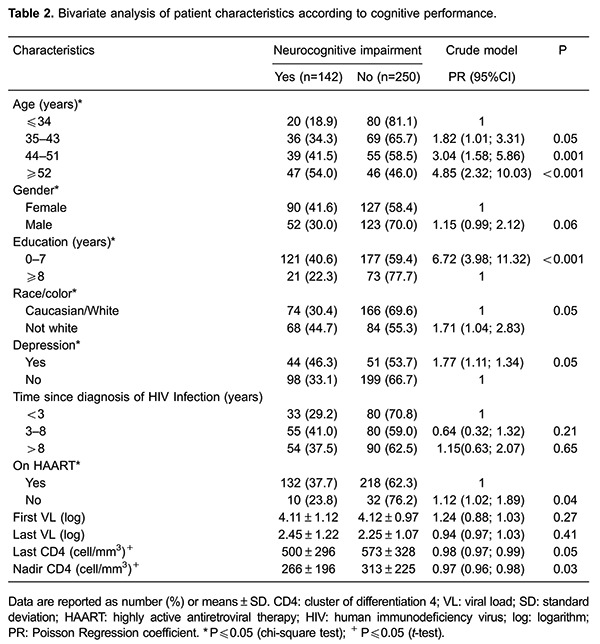

The regression analysis showed that age, educational level and skin color remained associated with NCI in HIV/AIDS patients at level 1. Patients aged 52 years or older were 4.85 times more likely (95%CI=2.34–10.03) to develop neurocognitive disorders compared with patients under 34 years of age. Individuals with less than eight years of education were 6.72 times more likely (95%CI=3.98–11.32) to develop neurocognitive disorders. Patients with a non-white skin color were 1.71 times more likely (95%CI=1.04–2.83) to develop subcortical disorders. After the other variables were adjusted by the variables from level 1, only depressive episodes remained associated (PR=1.96, 95%CI=1.12–3.42), whereas the others lost associative strength.

In addition, 89.4% of the patients in this sample were undergoing antiretroviral therapy, 52.3% used efavirenz (EFZ), 1.4% used nevirapine, and 35.7% used protease inhibitors. The mean and standard deviation of the length of EFZ use was 5.0±4.2 years. Forty percent of the patients undergoing antiretroviral therapy had started therapy more than 5 years earlier. The bivariate analysis indicated no association between the use of EFZ and cognitive impairment or depression.

## Discussion

The prevalence of HAND found in our study is in agreement with those obtained in other Brazilian studies (11,12) that used similar tools to diagnose HAND. In a prospective study (21) of 364 patients who underwent a full battery of neurocognitive tests, the prevalence of all forms of HAND ranged from 25 to 33% between 2007 and 2012. The prevalence obtained in our study, which included a simple battery of five tests, was similar. In another study (22) with a greater number of participants, including patients with other comorbidities, NCI was diagnosed in 58.5% of cases. In that study, the risk factors for symptomatic cognitive impairment in the HIV population were mainly the same as in the general population and NCI was not clearly associated with HIV-related factors. The factors associated with symptomatic cognitive impairment were depression, anxiety, low educational level and history of brain injury. The bivariate analysis conducted in this study showed an association between cognitive impairment and low educational level. A recent study (23) that monitored HIV-positive patients undergoing ART for 30 years confirmed these findings, showing a lack of association between neurocognitive loss and factors related to HIV infection. However, depressive symptoms were common, and cognitive impairment was also associated with traditional risk factors.

We found that the prevalence of cognitive impairment increased with age, a finding that is consistent with the results of other studies (24,25). In general, age is an important factor in the onset of NCI and is not necessarily related to HIV infection. The multicenter study by Wright et al. (13) demonstrated an association between cognitive impairment and cardiovascular risk factors in patients with higher CD4 counts and found no associations with variables directly associated with HIV infection. These factors are related to age and exhibit a higher prevalence in HIV-positive patients. In our study, we did not observe an association with cardiovascular impairment.

Among the factors directly related to HIV infection, a historically lower CD4 count (lowest CD4 count), a lower current CD4 count and the use of ART were associated with cognitive impairment in the bivariate analysis. These associations have been noted in other studies (5,11,14,24), and did not remain significant in the multivariate analysis, which may be due to a lack of power in our study or to the strong association of depression with our outcome. Cognitive impairment in the HIV-positive population remains frequent despite the use of ART and the reduction of neurological complications from immunosuppression (5,6). This finding may be related to factors directly associated with HIV or to multiple causes that are also observed in the general population (5,22,23). Determining the extent to which these disorders are secondary to HIV infection (HAND) according to American Association of Neurology criteria (2007) is complex and requires tools that are difficult to apply in clinical practice (8). Sacktor et al. (9) proposed the use of the IHDS as a useful tool for screening HIV-related dementia. However, those authors also noted several limitations of the IHDS: the tool is not useful for the diagnosis of mild cognitive impairment, it cannot be used to differentiate between varying degrees of HIV compromise, the effect of depression on the performance of this tool has not yet been determined, and it has a specificity of 55–57%. A recent systematic review (26) that evaluated the accuracy of the IHDS estimated a specificity of 55% and a sensitivity of 74% for the diagnosis of severe HAND and a sensitivity of 64% and a specificity of 66% for the diagnosis of all forms of symptomatic HAND. That review suggests that IHDS does not have an acceptable level of accuracy for HAND diagnosis and should not be used separately to distinguish between the different etiologies of cognitive impairment. In agreement with other studies (16,17,22,24,27,28), we found that depression was strongly associated with cognitive impairment and thus it can be an important confounding factor in the diagnosis of HAND when using tools such as the IHDS. When evaluating our patients, the diagnosis of HIV-related NCI may be overestimated if this factor is not considered.

The identification of depression in HIV patients is also important because of its association with more severe immunodeficiency, lower CD4 count, higher viral loads and more rapid disease progression. A greater decline in the CD4 count was associated with depression in males with HIV in an American cohort study (28). In a study (15) conducted in 1017 women in Uganda, where the prevalence of depressive symptoms was estimated to be 47%, the association of a CD4 count less than 50 with depression was evident. In another prospective study (29) with a four-year follow-up period, depression was associated with the evolution of the CD4 count and viral load. Patients with depression exhibited worse viral load control.

To identify psychiatric disorders, including depression, a useful tool used in this study was the MINI-Plus. The biometric characteristics of the MINI-Plus make this tool a good choice for use in daily clinical practice, in part due to the short time required for its implementation (20–30 min). The Portuguese form of MINI version 5.0 was found to be convenient for use in Brazil (20).

The limitations of this study include a moderate sample size, which limits the power for detecting associations, a modest test battery and the lack of local reference norms in Brazil. However, the use of only five instruments that can be easily and rapidly applied and that can detect prevalences similar to those obtained in studies using other more expensive and difficult to apply batteries could be considered an advantage in public health. In this sense, our study proposes an innovative approach for monitoring cognitive impairment in patients with HIV: the combination of the IHDS with practical tests previously used to diagnose neurocognitive changes caused by subcortical brain impairment (8,13,26). In this study, patients who presented poor performance in these tests and an IHDS score of 10 or less were classified as having NCI. The tests association aimed to increase the specificity of the instruments and was appropriate for evaluating the population studied. We propose that this strategy be subjected to further tests in future studies with a larger number of HIV patients and results compared with those of uninfected patients. Future studies should include a thorough neurocognitive assessment.

In conclusion, our findings confirmed a high prevalence of cognitive disorders in HIV-positive patients, and several factors are associated with these disorders. HAND diagnosis is difficult in daily clinical routines, and depression in these patients is associated with impairment, as determined through tests for the evaluation of cognitive impairment. The incorporation of easy to apply neurocognitive evaluation tools that are complementary to the IHDS and, just as important, the use of diagnostic and screening tools for evaluating depression in HIV/AIDS patients should be encouraged in daily clinical practice. Further studies are necessary to identify HIV-positive patients who would genuinely benefit from tests to identify HAND.
